# The mediation effect of peer relation and positive emotion between campus sports atmosphere and teenagers’ subjective well-being

**DOI:** 10.3389/fpsyg.2024.1471645

**Published:** 2024-12-18

**Authors:** Weibo Cheng, Long Jiao

**Affiliations:** ^1^School of Sports Science, Qufu Normal University, Qufu, China; ^2^Basic Education Group, Shandong Normal University, Jinan, China

**Keywords:** campus sports atmosphere, teenagers’ subjective well-being, peer relation, positive emotion, mediation

## Abstract

**Background:**

The healthy growth of young people affects the future of every country and nation. Their physical and psychological health problems have long been the focal point of every country. The purpose of this study is to investigate the influence of campus sports atmosphere on the teenagers’ subjective well-being.

**Method:**

This study was conducted among 10 to 15 year old teenagers in Jinan City, by means of campus sports atmosphere, peer relation, positive emotion and subjective well-being. The original data is exported in Excel format from Wenjuanxing Platform, and SPSS 26.0 is used to perform descriptive statistics, reliability, and correlation analysis.

**Results:**

The correlation coefficient of campus sports atmosphere, peer relations, positive emotion, and teenagers’ subjective well-being is significant; the results showed that the campus sports atmosphere had a positive predictive effect on the peer relationship (*β* = 0.699, *t* = 17.729, *p* < 0.01), and positive emotion (*β* = 0.38, *t* = 16.783, *p* < 0.01). Campus sports atmosphere can predict the teenagers’ subjective well-being positively (*β* = 0.289, *t* = 14.723, *p* < 0.01); The relationship between peer relations and positive emotion can predict the teenagers’ subjective well-being positively (*β* = 0.292, *t* = 15.24, *p* < 0.01; *β* = 0.355, *t* = 18.669, *p* < 0.01); Through Bootstrap Testing, we find that the campus sports atmosphere has a significant positive influence on the teenagers’ subjective well-being, which is 0.4969; The influence of the relationship between the campus sports atmosphere and the teenagers’ subjective well-being was significant, and the total indirect influence was 0.4352.

**Conclusion:**

The campus sports atmosphere not only influences teenagers’ subjective well-being directly, but also improves their well-being by the mediation of peer relations and positive emotion.

## Highlights

- The campus sports atmosphere not only influences teenagers’ subjective well-being directly, but also improves their well-being by the mediation of peer relations and positive emotion.
- The correlation coefficient of campus sports atmosphere, peer relations, positive emotion, and teenagers’ subjective well-being is significant.

## Introduction

The healthy growth of young people affects the future of each nation and country. Their physical and psychological health problems have long been the focal point of every country. The Chinese Ministry of Education reported in Dec. 2021 that the general health situation in elementary and middle schools is gradually improving, but the problems of obesity, visual impairment, mental illness, and chronic illness are still serious (Ministry of Education of the People’s Republic of China, 2021). Campus sports atmosphere is a kind of external atmosphere and environment which is recognized and recognized by the individual or community, and has a lasting and steady influence on all kinds of physical activities designed to improve the body, improve the health, and promote the overall development (Li and Zhang, 2018). The former research shows that the effective exercise can improve the subjective well-being of the individual, and the good campus sports atmosphere can stimulate the physical activity of the individual. Therefore, the campus sports atmosphere may be related to the well-being of teenagers. In view of the relationship between them, it is important to study the influence mechanism of campus sports atmosphere on teenagers’ subjective well-being and analyze their improving strategies.

Campus sports atmosphere has a positive positive effect on individual physical activity (Li et al., 2023). The good campus sports atmosphere is beneficial to the development of the teenagers’ sports habits, to the development of lifelong sports consciousness, and to make them feel the pleasure of exercise. Research has shown that a campus sports atmosphere can reduce student’s reliance on cell phone (Li and Dong, 2022) and negative feelings (Xu, 2022) by encouraging young people to exercise in order to improve their life satisfaction. Furthermore, physical training can be effective in improving interpersonal communication (Wang and Zhao, 2021), reducing negative feelings (Wang, 2019), enhancing resilience and improving subjective well-being (Ma et al., 2022). On this basis, we put forward the hypothesis H1: Campus sports atmosphere has positive predictive effect on teenagers’ subjective well-being.

Peer relationship is a kind of relationship that is built and developed in the course of communication among peers or individuals who have similar mental development. The relationship consists of meanings, expectations, and feelings created by a series of interactions (Zhou and Zhou, 2021). Good campus sports atmosphere is beneficial to build mutual relations and improve friendship quality (Li and Zhang, 2021). Poor peer relations may have an impact on the ability to adapt to society and academic performance (Dai and Peng, 2021), and may also increase the risk of internalising issues like depression and anxiety (Yan and Li, 2021). Moreover, it has been found that the relationship between parents and subjective well-being is partly mediated by peer relations (Zhang et al., 2019), and it is also indirectly affected by peer relations by means of academic achievement (Ma et al., 2019), emotion management, stress tolerance (Sun, 2023). On the basis of the above, this paper puts forward the hypothesis H2: The campus sports atmosphere can positively predict the subjective well-being of teenagers through the enhancement of the peer relations.

Positive emotion is an emotion related to happiness, which is generated by an individual as a consequence of both internal and external stimuli and events that meet their needs (Gu and Tong, 2021). A growing number of studies indicate that positive psychological interventions can reduce the depressive symptoms of pupils (Guo et al., 2015), regulate positive self-evaluation (Jiang et al., 2022), improve self-efficacy (Zhang and Wang, 2015) and subjective well-being (Zhang et al., 2017). These findings suggest that a good campus sports atmosphere can help the individual to feel happy and relaxed, thus removing the negative feelings. Meanwhile, positive emotion intervention can improve students’ subjective well-being. Therefore, this paper puts forward the hypothesis H3: The campus sports atmosphere can positively predict the subjective well-being of teenagers through the enhancement of the positive emotion.

**Figure 1:**
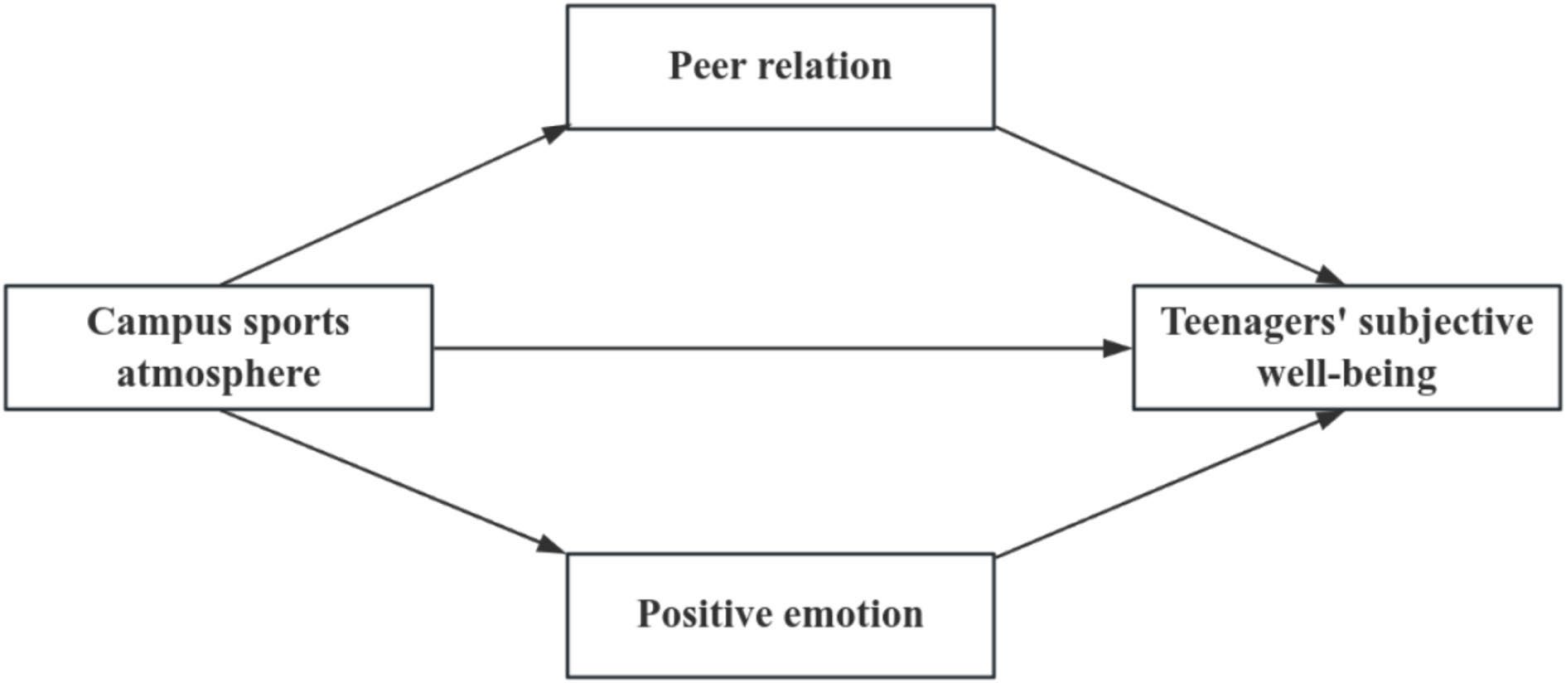
The proposed model.

Previous research has indicated that the campus sports atmosphere is related to the teenagers’ subjective well-being, and also the research on the relation between the peer relation and the teenagers’ subjective well-being. However, the present study is not concerned about the relationship between the campus sports atmosphere and the teenagers’ subjective well-being. Based on this, a hypothetical model (Figure 1) is proposed to study the influence of campus sports atmosphere on teenagers’ subjective well-being, and the mediation effect of peer relation and positive emotion. Based on the existing research, the effect mechanism among the variables is analyzed, and the effective optimization strategy is explored to improve teenagers’ subjective well-being.

## Methods

### Study population

In this research, a questionnaire survey was conducted among primary and secondary students from three elementary and secondary schools in Jinan City. Among teenagers from 10–15 years old, 1701 questionnaires were given out in grade 4, 5 and 6, and grade 1, 2 and 3. After excluding invalid questionnaires with low completion or arbitrary filling, 1,666 valid questionnaires remained, with an effective rate of 97.94%. Among them, there are 813 boys, 853 girls, 212 students in the fourth grade of primary school, 317 students in the fifth grade of primary school, 328 students in the sixth grade of primary school, 229 students in the first grade of junior high school, 322 students in the second grade of junior high school, and 258 students in the third grade of junior high school. Detailed guidance on the purpose of the survey, voluntary principles, and anonymous participation in the e-questionnaire guidance section should be provided, and the participants’ informed consent should be obtained.

### Measurement

#### Campus sports atmosphere scale

On the basis of Liu Weina and Zhou Chenglin’s revision of “Sports Atmosphere Scale,” the campus sports atmosphere scale consists of five dimensions: human relations, natural relations, information acquisition, interpersonal barriers, and conditional barriers (Liu et al., 2011). This scale is made up of 17 items and uses a 5-point scoring system. Scores from 1–5 represent “completely disagree” to “completely agree.” The higher the score, the better the campus sports atmosphere. The coefficient of Cronbach’s *α* was determined to be 0.85.

#### Subjective well-being scale

The subjective well-being scale adopts Campbell’s “Happiness Index Scale,” which includes two dimensions: “Overall Emotion Index” and “Life Satisfaction,” with a total of 7 items (Campbell, 1976). Using a 7-point scoring system, from “1” (least happy) to “7” (happiest), the higher the cumulative score on the scale, the stronger the subjective well-being. In this study, the coefficient of Cronbach’s *α* was determined to be 0.81.

#### Peer relationship scale

The Peer Relationship Scale, developed by Asher and revised by Zhang Yali, consists of three dimensions: Welcome, Rejection, and Loneliness (Zhang, 2008). There are 15 items in this scale, and the Likert 5-point scoring system is used, with scores ranging from “totally different” to “totally in agreement.” The higher the score, the better the peer relationship. The reverse scoring questions are: 2, 4, 6, 8, 9, 11, 12, 13, 14, 15. The coefficient of Cronbach’s *α* was determined to be 0.81.

#### Positive emotion scale

The Positive Affect and Negative Affect Scale was created by Watson and revised by Qiu et al. (2008). The Positive Affect Scale consists of 9 words describing emotion. Using the Likert 5-point scoring system (1 = very mild or no, 5 = very strong), the higher the score, the more positive the participants were. The coefficient of Cronbach’s *α* was determined to be 0.84.

The exploratory factor analysis yielded a KMO value of 0.73, and the Bartlett’s test of sphericity showed an approximate χ^2^ = 313.78, df = 3, *p* < 0.01. The confirmatory factor analysis results were as follows: χ^2^/df = 4.65 (*p* < 0.01), CFI = 0.93, GFI = 0.91, NFI = 0.92, TLI = 0.91, RMSEA = 0.05, and AVE = 0.92. The overall Cronbach’s α coefficient for the scale was 0.85, and the split-half reliability was 0.76.

## Statistical analysis

The original data is exported in Excel format from Wenjuanxing Platform, and SPSS 26.0 is used to perform descriptive statistics, reliability, and correlation analysis. All data were processed using this software. The PROCESS Macro Program 4, which was developed by the Hayes group, was used to analyze the mediation effects of peer relationship and positive emotion. A sample correction was performed 5,000 times, and a 95% CI was calculated for the intermediate effect. If the 95 per cent confidence interval is not zero, the direct or indirect effects are deemed to be relevant.

Since all the data in this study are derived from a subjective questionnaire, there are likely to be some common methodological errors. Harman Single-Factor Test was adopted in the common method bias testing. Among them, 12 of them were more than 1, and the first factor accounted for 39.46% of the variance, which was below the 40% critical criterion suggested by Podsakoff.

## Results

### Descriptive analysis

Based on the analysis of the original data, we selected 1,666 questionnaires, and found that the average annual income of 69.6% of the sample households (Table 1). 86.2% of adolescent participants scored “average” or higher in subjective well-being, and 21.7% were “happiest.” Of those surveyed, 91.1% thought that the campus sports atmosphere was “average” or higher, and only 0.6% thought that the sports atmosphere in their campus was bad. The average score of peer relationship and positive emotion was 3.68 and 3.75, respectively. The results showed that there were no significant differences in the sex of the main variables, such as the campus sports atmosphere and the teenagers’ subjective well-being. Based on one way ANOVA method, we found that there were significant differences in age and family income between the two groups (*F* = 2.675, *p* = 0.03; *F* = 4.187, *p* = 0.002).

**Table 1:** Descriptive statistical results of each variable.

Variables		Variables	
Family income		Subjective well-being	
Low income	220 (13.2%)	Most unhappy	8 (0.5%)
Middle income	1,159 (69.6%)	Very unhappy	52 (3.1%)
High income	287 (17.2%)	Relatively unhappy	169 (10.1%)
Campus sports atmosphere		Average	284 (17%)
Very poor	10 (0.6%)	Relatively happy	359 (21.5%)
Relatively poor	139 (8.3%)	Very happy	433 (26%)
Average	351 (21.1%)	Most happy	361 (21.7%)
Good	641 (38.5%)	Peer relation	3.68 ± 0.79
Very good	525 (31.5%)	Positive emotion	3.75 ± 0.82

### Correlation analysis

Pearson correlation analysis was carried out on the major variables by SPSS (Table 2). There was a significant positive correlation between the teenagers’ subjective well-being and their campus sports atmosphere, their peer relationship and their positive emotion (*r* = 0.542, 0.523, 0.561; *p* < 0.01). The atmosphere of campus physical exercise is significantly positively correlated with peer relationships, as well as with positive emotions (*r* = 0.401, 0.384; *p* < 0.01). The results indicated that the better the campus sports atmosphere, the better the peer relations, the stronger the positive.

**Table 2:** Correlation analysis results of major variables.

Variables	1	2	3	4
Campus sports atmosphere	1			
Subjective well-being	0.542**	1		
Peer relation	0.401**	0.523**	1	
Positive emotion	0.384**	0.561**	0.325**	1

*p < 0.05, **p < 0.001.

### Multiple regression analysis

The SPSS Process Component Model 4 was used for multiple regression analysis. After adjusting for sex, age and family income, the campus sports atmosphere was used as an independent variable, and the relationship between peer relation and positive emotion was used as the dependent variable. The results of regression analysis indicated that the campus sports atmosphere had significant positive predictive effect on the peer relationship (*β* = 0.699; *p* < 0.01), and positive emotion (*β* = 0.38; *p* < 0.01). Campus sports atmosphere, peer relations, positive emotion were used as independent variables, and teenagers’ subjective well-being was taken as dependent variable. The result of regression analysis indicated that the campus sports atmosphere could predict the teenagers’ subjective well-being significantly (*β* = 0.289; *p* < 0.01), and the relationship between peer relation and positive emotion was positively predicted (*β* = 0.292; *p* < 0.01; *β* = 0.355; *p* < 0.01) (Table 3).

**Table 3:** Multiple regression analysis results of the main variable relationships.

Regression equation		Overall fitting index		Significance of regression coefficient	
Dependent variable	Predictive variables	*R^2^*	*F*	*β*	*t*
M1	SEX	0.164	81.175^**^	−0.007	−0.325
	AGE			−0.027	−1.215
	INC			0.038	1.695
	X			0.699	17.729^**^
M2	SEX	0.150	73.270	−0.024	−1.064
	AGE			−0.010	−0.447
	INC			0.046	2.019^*^
	X			0.380	16.783^**^
Y	SEX	0.509	287.130^**^	−0.009	−0.538
	AGE			0.031	1.809
	INC			−0.002	−0.096
	M1			0.292	15.240^**^
	M2			0.355	18.669^**^
	X			0.289	14.723^**^

X is the independent variable: campus sports exercise atmosphere, M1 is the mediating variable: peer relationship, M2 is the mediating variable: positive emotions, Y is the dependent variable: adolescent happiness, SEX is gender, AGE is age, INC is annual household income. *p < 0.05, **p < 0.001.

### Mediation effect analysis

Through regression analysis, we found that the relationship of peer relation and positive emotion had a positive influence on teenagers’ subjective well-being. Thus, the mediation effect was tested with the nonparametric Percentile Bootstrap Method, with 5,000 replicates and 95% CI (Table 4). First of all, this paper analyses and tests the relationship between campus sports atmosphere and teenagers’ subjective well-being. The direct effect of campus sports atmosphere on teenagers’ subjective well-being was 0.4969, and the overall effect was 53.32%. The Bootstrap 95% CI does not contain 0, which suggests that the campus sports atmosphere has a significant impact on teenagers’ subjective well-being. Secondly, this paper analyses and tests the mediation effect of peer relation and positive emotion on campus sports atmosphere and teenagers’ subjective well-being. The indirect effects of peer relation and positive emotion on teenagers’ subjective well-being were 0.201 and 0.2342 respectively, and their Bootstrap 95% CI was not included. Young people who are in good campus sports atmosphere can improve their subjective well-being through improving their relationship with their peers or improving their positive mood.

**Table 4:** Direct and intermediate test results.

Paths	Effect	SE	95%CI		%
			LLCI	ULCI	
Total effect	0.932	0.0354	0.8626	1.0015	100%
Direct effects	0.4969	0.0337	0.4307	0.5631	53.32%
Total indirect effect	0.4352	0.0286	0.38	0.4911	46.70%
X-M1-Y	0.201	0.0173	0.1675	0.2359	21.57%
X-M2-Y	0.2342	0.0219	0.1926	0.2774	25.13%

**Figure 2:**
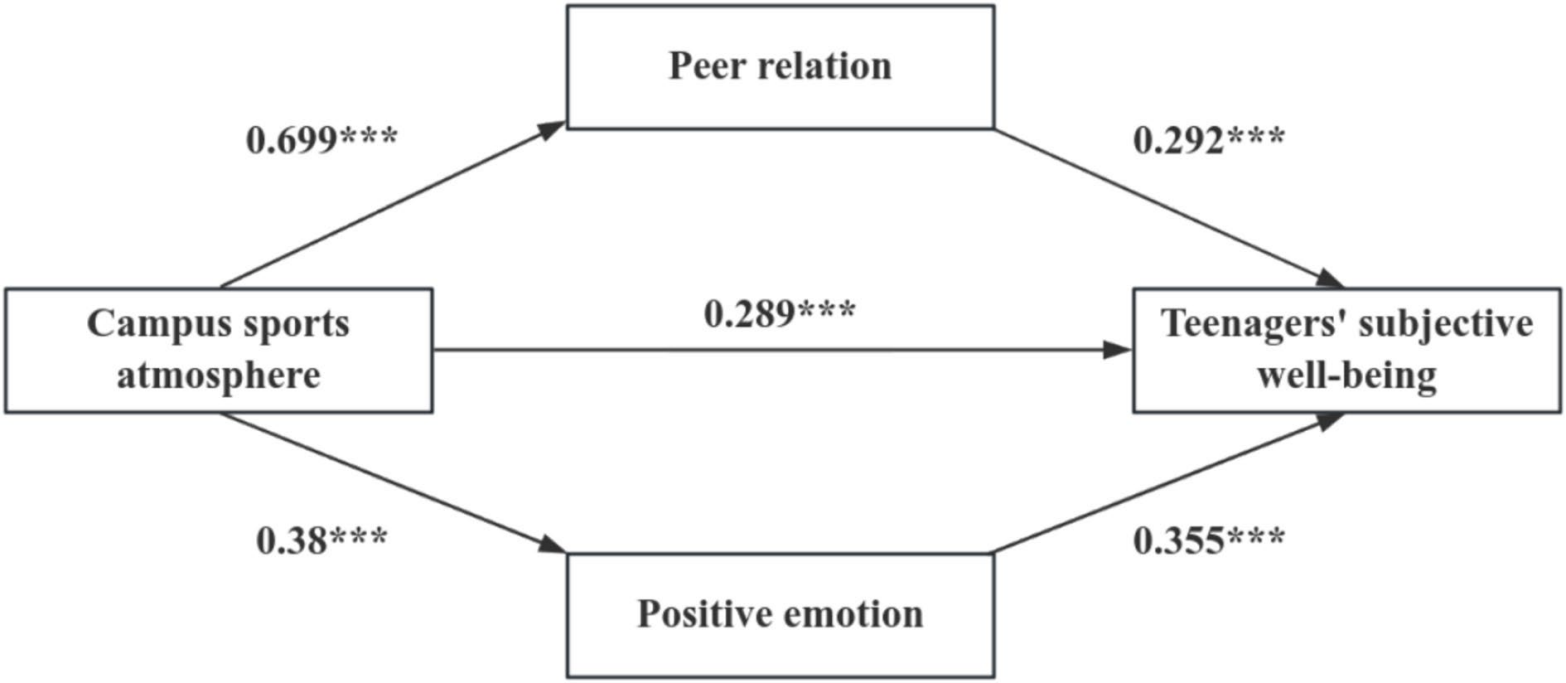
The final mediation model.

Finally, the result of the integration of the peer relation and the positive emotion test was 0.4352, which was 46.70%. Furthermore, by comparing the mediation effects of peer relation and positive emotion, we find that the mediation effect of the peer relationship is −0.0332, which shows that in the influence of campus sports atmosphere on the teenagers’ subjective well-being, the positive emotion is more effective than the peer relationship (Figure 2). Therefore, it is more effective to promote the development of positive emotion by creating a campus sports atmosphere and improving the teenagers’ subjective well-being.

## Discussion

In this research, the influence of campus sports atmosphere, peer relations and positive emotion on teenagers’ subjective well-being was analyzed. This paper makes an in-depth study on the relationship between the campus sports atmosphere and the students’ subjective well-being, which can be used to help them grow up healthily and happily.

The findings of this study provide strong support for the positive relationship between the campus sports atmosphere and teenagers’ subjective well-being, thereby confirming Hypothesis H1. This result aligns with existing literature indicating that a conducive physical exercise environment can enhance the happiness and well-being of adolescents (Xiao, 2013). The establishment of a positive campus sports atmosphere plays a crucial role in reducing feelings of anxiety and depression (Zhang and Zhou, 2013), fostering positive emotional experiences, and serving as a significant motivator for the promotion of sustained physical activity (Chen et al., 2006). Consequently, it is essential for educators and administrators to prioritize the creation of a supportive campus sports culture. By doing so, they can increase student participation in physical activities, alleviate academic pressures, and cultivate positive emotions among the youth.

Moreover, it is critical to improve the security of school sports facilities, offer professional guidance outside of class, implement a robust management system, and introduce relevant policies to support the development of safe, diversified, and scientifically-backed physical activity programs for students. Drawing on international examples, such as Finland’s “School Sports Plan,” which emphasizes the establishment of a healthy school sports culture as one of its three core pillars (Yang et al., 2022), could also be beneficial. Finnish research demonstrates that a positive sports environment not only enhances children’s motor skills (Zeng and Wang, 2017) but also has significant benefits for academic performance (Liu and Wang, 2011), health behaviors (Cao et al., 2017), and attention (Blom et al., 2018). Furthermore, over a third of Finnish children engage in regular physical activity and develop healthy sleep patterns (Kaapa et al., 2022). By integrating these insights with China’s unique educational and cultural context, we can further enhance the campus sports atmosphere to promote the healthy and happy development of adolescents.

The study also reveals the mediating role of the campus sports atmosphere in the relationship between physical activity and subjective well-being, supporting Hypothesis H2. This finding underscores the notion that a positive campus sports environment reflects the presence of healthy sports habits and attitudes among peers, which, in turn, influence individuals’ engagement in sports activities. As individuals are unconsciously affected by their surroundings, this environment encourages greater participation in physical activities, which not only improves personal fitness but also strengthens peer relationships (Zhang and Xu, 2022). Strengthening emotional bonds through shared physical activities has been shown to reduce the risk of depression and anxiety (La Greca and Harrison, 2005) and decrease the likelihood of problematic behaviors (Jin et al., 2012). Thus, the better the campus sports atmosphere, the stronger the sense of community and mutual support among students, ultimately leading to enhanced subjective well-being. To capitalize on this, it is important to implement group-based physical education programs that foster cooperation and teamwork, enabling students to build positive relationships, relieve academic stress, and, ultimately, improve life satisfaction.

Furthermore, the results highlight the role of positive emotions as an additional mediating variable in influencing teenagers’ subjective well-being, supporting Hypothesis H3. A well-developed campus sports atmosphere can positively impact students’ mood, a finding consistent with previous research. Elements such as the physical environment, social support, the nature of the exercise, and the content of the activities (Chen et al., 2006) all contribute to shaping adolescents’ subjective experiences. Engaging in physical activities allows students to cope with negative emotions and enhance positive feelings and life satisfaction (Dong et al., 2022), thus boosting their overall well-being. These findings suggest that a more vibrant and supportive campus sports atmosphere not only elevates students’ mood but also leads to improved subjective well-being over time. To facilitate this, schools should encourage youth participation in physical activities, as regular engagement in exercise has been shown to promote emotional stability and well-being (Zhao and Huang, 2023).

## Conclusion

In this research, a group of 10 to 15 years old teenagers was chosen to analysis the influence of campus sports atmosphere on teenagers’ subjective well-being, and the mediation effect of peer relation and positive emotion. Research has found that campus sports atmosphere not only has direct effect on teenagers’ subjective well-being, but also improves their well-being through the mediation of peer relationship and positive emotion.

This study reveals the impact of campus sports atmosphere on teenagers’ subjective well-being, but there are still some limitations. First, the sample was drawn from teenagers aged 10 to 15 in Jinan City, and the regional and age-specific limitations may affect the generalizability of the results. Future research could include samples from different regions and age groups to enhance external validity. Second, the use of self-reported questionnaires may introduce subjective bias, so future studies could incorporate other data collection methods, such as observation or interviews, to enhance the robustness of the findings. Moreover, as this study used a cross-sectional design, it cannot establish causal relationships. Future research could adopt longitudinal or experimental designs to validate the causal pathways between variables.

This study expands the theoretical framework of the relationship between campus sports atmosphere and teenagers’ subjective well-being, showing that sports atmosphere not only directly affects well-being but also exerts an indirect effect through peer relationships and positive emotions. This finding holds significant implications for psychology and education, enriching the understanding of how physical activity influences adolescent mental health. Future research could explore other potential mediators, such as self-esteem and self-efficacy, to further elucidate the mechanisms linking campus sports atmosphere and subjective well-being. Additionally, cross-cultural research could help understand how cultural differences affect this relationship, warranting validation in different countries and regions. With the development of big data and artificial intelligence, future studies can employ these emerging technologies for dynamic analysis, offering more precise strategies to enhance adolescent well-being.

## Data Availability

The original contributions presented in the study are included in the article/supplementary material, further inquiries can be directed to the corresponding author.

## References

[ref1] Blom A. Tammelin T. Laine K. Tolonen H. (2018). Bright spots, physical activity investments that work: the Finnish schools on the move programme. Br. J. Sports Med. 52, 820–822. doi: 10.1136/bjsports-2017-097711, PMID: 28954798 PMC6029642

[ref2] Campbell A. (1976). Subjective measures of well-being. Am. Psychol. 31, 117–124. doi: 10.1037/0003-066X.31.2.117 1267244

[ref3] Cao Z. B. Chen P. J. Zhuang J. Liu Y. Wang L. Han J. (2017). History of sport health policy in developed countries and revelation for the healthy China. China Sport Sci. 37, 11–23. doi: 10.16469/j.css.201705002

[ref4] Chen S. P. Li S. Z. Yan Z. L. (2006). Research on mechanism of exercise persistence based on sport commitment theory. China Sport Sci. 12, 48–55. doi: 10.16469/j.css.2006.12.010

[ref5] Dai B. R. Peng M. (2021). The relationship between peer friendship quality and social adaptability of rural left-behind children: a moderated mediation analysis. J. Psychol. Sci. 44, 1361–1368. doi: 10.16719/j.cnki.1671-6981.20210611

[ref6] Dong Y. Q. Ge Y. Y. Ding F. Zhong J. W. Li X. C. (2022). Influence of cumulative ecological risk on college students’ physical exercise: the mediating effect of exercise atmosphere and exercise self-efficacy. Chin. J. Health Psychol. 8, 1244–1249. doi: 10.13342/j.cnki.cjhp.2022.08.025

[ref7] Gu Y. Tong H. J. (2021). A review of the regulating effect of meditation on positive emotions. Med. Philos. 42, 48–53.

[ref8] Guo Y. F. Li S. W. Chen O. Y. Zhang Q. H. Qi X. J. Zhang J. P. (2015). An 8-week group positive psychotherapy intervention on depression associated self-efficacy and subjective well-being in female nursing students. Chin. Ment. Health J. 29, 172–177.

[ref9] Jiang Y. Z. Bai X. L. Zhang L. Zhao S. Q. (2022). The relationship between Adolescents' online positive self-presentation and well-being: the role of online positive feedback and self-esteem. Psychol. Dev. Educ. 38, 45–53. doi: 10.16187/j.cnki.issn1001-4918.2022.01.06

[ref10] Jin C. C. Liu Y. Chen L. (2012). The effect of negative social environment on problem behavior of unattended and migrated children: parent-child relationship and peer relationship as moderators. J. Psychol. Sci. 35, 1119–1125. doi: 10.16719/j.cnki.1671-6981.2012.05.003

[ref11] Kaapa M. Palomaki S. Fedewa A. Valleala U. M. Hirvensalo M. (2022). The role of parental support and the Students' opinions in active Finnish physical education homework. Int. J. Environ. Res. Public Health 19:11924. doi: 10.3390/ijerph191911924, PMID: 36231254 PMC9565897

[ref12] La Greca A. M. Harrison H. M. (2005). Adolescent peer relations, friendships, and romantic relationships: do they predict social anxiety and depression? J. Clin. Child Adolesc. Psychol. 34, 49–61. doi: 10.1207/s15374424jccp3401_5, PMID: 15677280

[ref13] Li H. Dong B. L. (2022). Relationship between school climate, Mobile phone addiction and adolescent physical exercise:a 3-year longitudinal follow-up survey. J. Tianjin Univ. Sport 37, 152–159. doi: 10.13297/j.cnki.issn1005-0000.2022.02.005

[ref14] Li N. Wang D. Zhao X. Li Z. (2023). Effect of leisure exercise atmosphere on smartphone addiction among college students: the mediator role of negative emotion and self-control aschain mediator. China J. Health Psychol. 31, 288–294. doi: 10.13342/j.cnki.cjhp.2023.02.022

[ref15] Li Y. Zhang J. (2018). Research on the way of cultivating physical training atmosphere on campus. Sports Cult. Guide. 3, 113–117.

[ref16] Li S. J. Zhang Q. L. (2021). Influence of teaching mode of partner physical education on the friendship quality of primary school students in grade 3-4. J. Shandong Sport Univ. 37, 69–74. doi: 10.14104/j.cnki.1006-2076.2021.04.009

[ref17] Liu X. H. Wang H. B. (2011). The education system with the least gap: the current situation, characteristics, and implications of early childhood education in Finland. Prim. Sec. School. Abroad 9, 32–37.

[ref18] Liu W. N. Zhou C. L. Sun J. (2011). The influence of adolescent outdoor exercise motivation on exercise persistence: the mediating role of exercise atmosphere. China Sport Sci. 31, 1409–1410. doi: 10.16469/j.css.2011.10.006

[ref19] Ma B. B. Dai W. J. Li C. N. (2019). Migrant adolescents’ interpersonal relationship and subjective well-being: the mediating effect of academic burnout and academic engagement. Chin. J. Spec. Educ. 12, 63–71.

[ref20] Ma C. Shi Z. G. Wang X. L. Tian Y. G. (2022). Influence of physical activity on college students subjective well- being: the mediating effect of peer relationship and self- cognition. Chin. J. Health Psychol. 30, 893–899. doi: 10.13342/j.cnki.cjhp.2022.06.020

[ref21] Ministry of Education of the People’s Republic of China. The Ministry of Education held a press conference to introduce the effectiveness of the "I do practical things for the masses" activity around strengthening the "five management" work in primary and secondary schools (2021). Available at: http://www.moe.gov.cn/fbh/live/2021/53908/twwd/202112/t20211222_589329.html

[ref22] Qiu L. Zheng X. Wang Y. F. (2008). Revision of the positive affect negative affect scale. Chin. J. Appl. Psychol. 14, 249–254.

[ref23] Sun W. Y. (2023). Interpersonal relationship in school and well-being in primary and secondary school students: the mediating role of emotional regulation. Chin. J. Health Psychol. 31, 148–156. doi: 10.13342/j.cnki.cjhp.2023.01.026

[ref24] Wang F. B. H. (2019). Family capital and parenting style: family class differences in adolescent physical activity. China Sport Sci. 39, 70–75. doi: 10.16469/j.css.201903006

[ref25] Wang J. Zhao Y. P. (2021). Influence of physical exercise on psychological happiness: mediation function of self-efficacy and regulating function of exercise atmosphere. J. Phys. Educ. 37, 70–75. doi: 10.16419/j.cnki.42-1684/g8.2021.03.011

[ref26] Xiao Y. W. (2013). The influencing factors of physical exercise on the Happimess index of the Henan University graduate. Master's Thesis. Henan: Henan Normal University.

[ref27] Xu C. (2022). The mechanism of college sports atmosphere on Students’Life satisfaction. J. Jilin Sport Univ. 37, 152–159.

[ref28] Yan Z. Q. Li S. (2021). The Association of Empathy on depression: the moderating effect of peer-relationship. Stud. Psychol. Behav. 19, 424–430.

[ref29] Yang Y. G. Wang X. Z. Kong L. (2022). Reference and relief: characteristics and inspirations of “Finnish schools on the move”. J. Chengdu Sport Univ. 48, 113–120. doi: 10.15942/j.jcsu.2022.03.018

[ref30] Zeng Y. S. Wang J. (2017). The development, features and enlightenment of school physical education in Finland. J. Chengdu Sport Univ. 43, 121–126. doi: 10.15942/j.jcsu.2017.06.020

[ref31] Zhang Y. L. (2008). Research on self concept and school adaptation of junior high school students. Xi'an: Northwest University.

[ref32] Zhang X. X. Guo H. Y. Lin D. H. (2019). A study on the relationship between parent-child, peer, teacher-student relations and subjective well-being of adolescents. Psychol. Dev. Educ. 35, 458–466. doi: 10.16187/j.cnki.issn1001-4918.2019.04.09

[ref33] Zhang P. Wang H. B. (2015). Mediating effect of regulatory emotional self-efficacy on relationships among neuroticism, extraversion and subjective well-being in college students. Chin. Ment. Health J. 29, 139–144.

[ref34] Zhang L. R. Wu X. F. Yang S. Q. Bie S. L. Chen C. (2017). The impact of positive psychology courses on subjective well-being of college students. Chin. J. School Health 38, 1409–1410. doi: 10.16835/j.cnki.1000-9817.2017.09.040

[ref35] Zhang D. Xu J. F. (2022). Community environment and physical exercise: an empirical analysis based on Clds2018 China sport. Science 42, 88–97. doi: 10.16469/j.css.202201008

[ref36] Zhang R. R. Zhou C. L. (2013). Emotional change and short-term emotional benefits of a single bout of physical activity. China Sport Sci. 33, 52–61. doi: 10.16469/j.css.2013.01.009

[ref37] Zhao J. Huang L. H. (2023). Influence of physical exercises on pilot students’ emotional stability: chain mediation effects of perceived social support and self-efficacy. Chin. J. Health Psychol. 31, 446–451. doi: 10.13342/j.cnki.cjhp.2023.03.024

[ref38] Zhou G. D. Zhou G. M. (2021). Peer relationship and Children’s reactive and proactive aggression: a cross-lagged regression analysis. Stud. Psychol. Behav. 19, 620–627.

